# Impact of financial deprivation in first-episode psychosis: a prospective 4-year follow-up study

**DOI:** 10.1007/s00127-025-03006-y

**Published:** 2025-10-17

**Authors:** Christy Lai Ming Hui, Eddie Chi Yuen Lui, Charlie Cheuk Lam Wong, Eunice Man Hei Choi, Nicole Cai Lin Yang, Yi Nam Suen, Edwin Ho Ming Lee, Sherry Kit Wa Chan, Wing Chung Chang, Eric Yu Hai Chen

**Affiliations:** 1 Department of Psychiatry, School of Clinical Medicine, Li Ka Shing Faculty of Medicine, University of Hong Kong, Hong Kong, China; 2 School of Nursing, Li Ka Shing Faculty of Medicine, University of Hong Kong, Hong Kong, China; 3 State Key Laboratory of Brain and Cognitive Sciences, University of Hong Kong, Hong Kong, China; 4 Orygen National Center of Excellence in Youth Mental Health, University of Melbourne, Melbourne, Australia

**Keywords:** Psychiatry, Schizophrenia, Deprivation, Outcome, Risk factors

## Abstract

**Purpose:**

Socioeconomic disadvantage can exacerbate psychosis outcomes, yet the long-term impact of financial deprivation remains underexplored. This study examined how financial deprivation at first-episode psychosis affects clinical, functional, and neurocognitive outcomes over four years.

**Methods:**

Participants were 240 adults (26–55 years) from the Jockey Club Early Psychosis project in Hong Kong, China. Financial deprivation was defined as household income < 50% of the median for household size. Regression analyses assessed the predictive value of baseline financial deprivation on four-year outcomes, controlling for sociodemographic and premorbid confounders. Age-subgroup analyses explored differential effects across developmental periods (< 33, 33–42, > 42 years).

**Results:**

At baseline, 121 participants (51.7%) were financially deprived, characterized by lower education, more siblings, foreign-born status, and residence in public housing. Baseline financial deprivation predicted worse four-year clinical, functional, and quality of life outcomes four years later. Financial hardship did not appear to predict neurocognitive outcomes at follow-up. Age-subgroup analyses indicated the strongest and most consistent effects in participants > 42 years, with minimal to modest effects in younger subgroups.

**Conclusions:**

Financial deprivation during early psychosis represents a high-risk subgroup, particularly in later adulthood, with persistent symptomatic and functional impairments. Early identification and age-sensitive interventions, such as vocational support, social benefits assistance, and programs promoting social integration and independent living, are essential. Policy measures targeting socioeconomic disadvantage may mitigate long-term impacts on illness trajectory and recovery.

**Supplementary Information:**

The online version contains supplementary material available at 10.1007/s00127-025-03006-y.

## Introduction

The development and course of psychosis-spectrum disorders are shaped by a range of biological and environmental factors, with growing evidence highlighting the significant influence of social and financial disadvantage. A systematic review of 28 studies reported a higher risk and prevalence of psychosis-spectrum disorders in more deprived areas, though the authors also noted that some studies found non-significant results after adjusting for individual and neighborhood-level factors [1]. These findings underscore the complex and context-dependent relationship between financial deprivation and the onset and outcomes of psychotic disorders. Low income may also hinder recovery through reduced social support, increased exposure to negative life events [2], and restricting access to quality treatment, medications, education, and employment opportunities [3].

### Limitations in current evidence on financial deprivation and psychosis recovery

The long-term impact of financial deprivation on recovery from psychosis remains unclear. Most studies rely on short-term or cross-sectional designs. For example, a cross-sectional study in Canada found that individuals with schizophrenia and lower incomes reported more severe psychopathology (Brief Psychiatric Rating Scale: 42.2 vs. 37.4) and reduced psychosocial functioning (Social and Occupational Functioning Assessment Scale: 59.3 vs. 63.5) [4]. Similarly, a study conducted in the United States (US) reported that higher parental occupational level, a proxy for household income, was associated with fewer positive symptoms at 6-month follow-up (adjusted OR = 4.88 for hallucinations and 2.46 for delusions) [5]. Furthermore, while research in East Asian settings is limited, preliminary findings suggest that financial deprivation is linked to an increased risk of schizophrenia at the provincial level (OR = 1.03) [6] and poorer clinical and functional outcomes in women with first-episode psychosis (FEP) [7].

Further, prior studies often lack comprehensive measurement across symptomatic, psychosocial, and cognitive domains. For instance, a long-term follow-up study in rural China assessed only unemployment rates and symptom severity [8], without including neurocognitive functioning or quality of life assessments [9], obscuring how financial deprivation may affect multiple aspects of recovery after FEP.

### Bidirectionality and potential confounding in financial deprivation and psychosis outcomes

Financial hardship may act as a chronic stressor [10, 11], contributing to social withdrawal, interpersonal sensitivity, and emotional dysregulation, thereby increasing the risk for psychosis onset [12, 13]. Conversely, psychosis onset and its lived experience may lead to downward social mobility and subsequent financial hardship [14, 15]. Longitudinal evidence provides some clarity on this relationship: Richardson and colleagues reported that financial difficulties predicted later symptoms of psychosis and distress, but symptoms did not predict subsequent financial strain [13]. Nonetheless, beyond bidirectionality, other sociodemographic and contextual factors, such as education, housing, and immigrant status, are well-established predictors of psychosis outcomes [16, 17] and are themselves linked to socioeconomic disadvantage [18, 19]. These factors may confound or mediate the relationship between financial deprivation and illness trajectories.

### The current study

Recognizing these complexities, the present study examines financial deprivation as a distinct indicator of socioeconomic disadvantage while accounting for key confounding variables. Our aim was to clarify whether financial hardship independently contributes to psychosis outcomes in Hong Kong, a setting characterized by pronounced wealth inequality (Gini coefficient = 0.54 in 2016) [20], high poverty rates (up to 23.6%) [21], and a fragmented social safety net, particularly in preventive care and timely access to medical support [22]. Given the scarcity of research on financial deprivation and psychosis outcomes in East Asia, Hong Kong represents a unique context to examine these dynamics. Specifically, this prospective longitudinal cohort study investigates the impact of financial deprivation on clinical, functional, and neurocognitive outcomes over four years, adjusting for key covariates, and explores whether these effects differ across developmental periods by conducting age-stratified subgroup analyses. Together, this study will provide context-specific insights into how socioeconomic disadvantage may shape illness trajectories in a high-inequality, East Asian urban environment.

## Methods

### Study design and participants

360 adults experiencing FEP were consecutively recruited from June 2009 to August 2011 as part of a 4-year randomized controlled trial under the Jockey Club Early Psychosis (JCEP) project in Hong Kong, China. While most early intervention (EI) services target individuals ≤ 35 years [23–25], later-onset cases are well-documented but under-researched. Following evidence of distinct profiles between adolescent- and adult-onset psychosis [26], the JCEP project was designed to include individuals aged 26–55 years, extending EI research to older adults with psychosis [27, 28].

Participants were randomized to receive either: (1) two years of EI followed by two years of standard care (*n* = 120), (2) four years of EI (*n* = 120), or (3) four years of standard care (*n* = 120). Participants who did not receive EI (*n* = 120) were excluded from the analysis to better isolate the impact of financial deprivation on psychosis recovery outcomes, as a previous study found a significant impact of EI on clinical and functional outcomes [28].

**Fig. 1:**
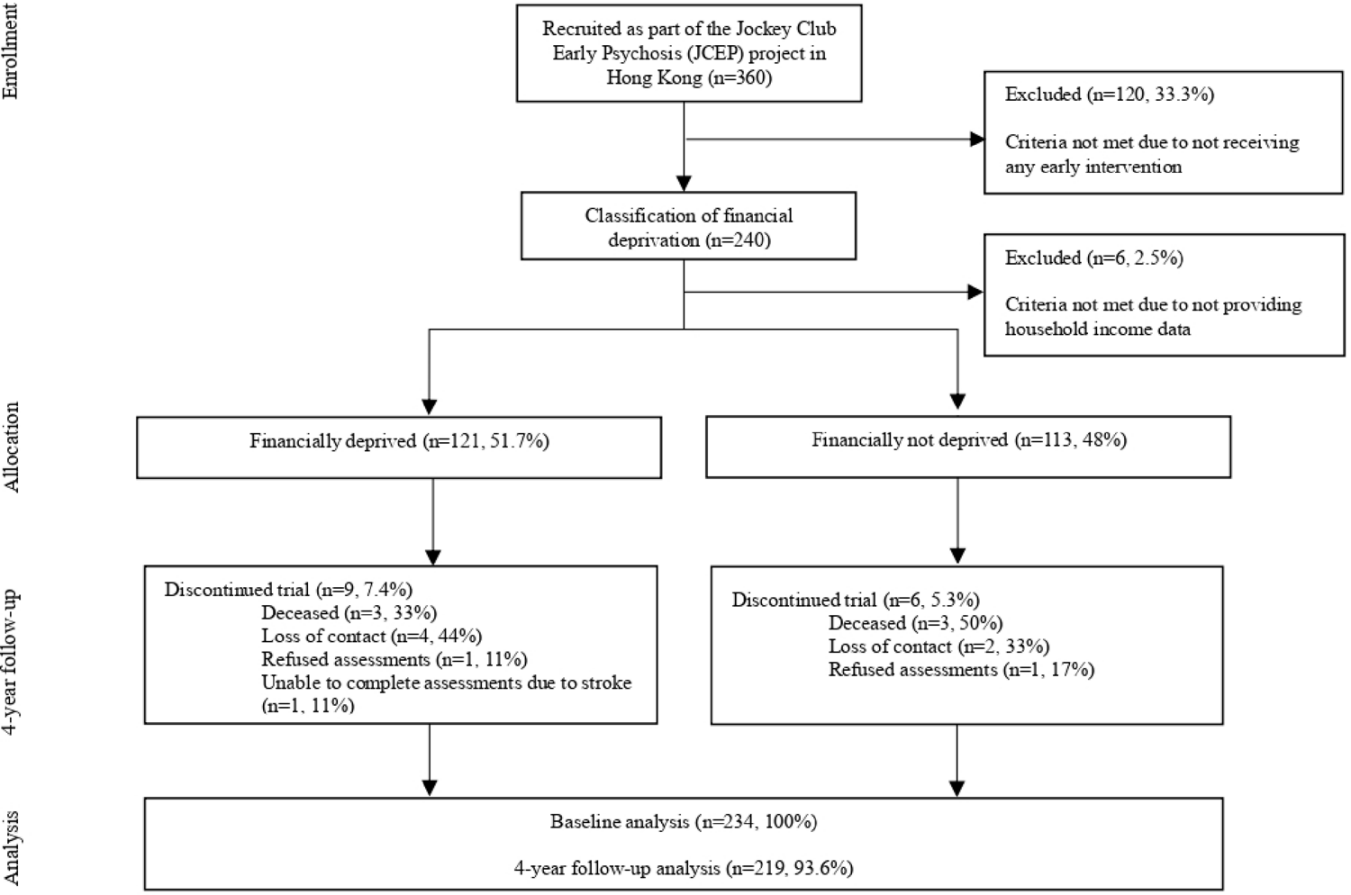
Flow of participants through the study. Participants were categorizes into financially deprived group (121, 51.71%) and financially not deprived group (113, 48.3%). At 4-year follow-up, 15 patients (6.4%) lost to follow up due to decease (n = 6), refused assessment (n = 2), loss of contact (n = 6) and stroke (n = 1)

Clinical, functional, and neurocognitive outcomes were measured at baseline and at four years (Fig. 1). Financial deprivation was defined using Hong Kong’s official poverty line, based on household income below “50% of median monthly household income” for a given household size [29]. For example, individuals living alone with a monthly income below HKD $3600 (the poverty line for a single-person household) would be classified as financially deprived (refer to **Online Resource 1** for details). As such, participants were categorized into two groups: financially deprived and not financially deprived.

The inclusion criteria were: (1) Cantonese-speaking Chinese adults, (2) aged 26–55, (3) diagnosed with various psychosis-spectrum disorders (specified according to DSM-IV [30]), (4) diagnosed with FEP with no more than 12 months of prior antipsychotic treatment following onset. The exclusion criteria were: (1) organic brain conditions, (2) intellectual disability, (3) substance-induced psychosis, or (4) risk of suicidal or violent behavior.

The study has been approved by the institutional review boards and carried out in accordance with the principles of Good Clinical Practice and the Declaration of Helsinki. Written informed consent was obtained from all participants.

### Measures

#### Basic demographics and premorbid measures

Baseline assessments were conducted upon entry into the project. Demographic information, including age, gender, place of birth, years of education, marital status, employment status, duration of untreated psychosis (DUP), mode of onset, psychiatric diagnosis, medication (type, dosage, and compliance), family history of psychiatric disorders, and monthly household income were collected. Stressful life events in the past six months were recorded by the List of Threatening Experiences [31]. Premorbid functioning and premorbid personality were evaluated with the Premorbid Adjustment Scale (PAS) [32] and the Premorbid Schizoid-Schizotypal Traits scale (PSST) [33]. Help-seeking characteristics were assessed with the Pathways to Care in Psychosis instrument [34].

#### Clinical, functional, and neurocognitive outcomes

Assessments of clinical, functional, and neurocognitive functioning were evaluated at baseline and at four years. All assessments were administered by trained research assistants who underwent intensive training in the use of study instruments and have been used or validated in Chinese individuals with psychosis [35–40].

Positive and negative symptoms were measured using the Positive and Negative Syndrome Scale (PANSS) [41], Scale for Assessment of Positive Symptoms (SAPS) and Scale for Assessment of Negative Symptoms (SANS) [42]. Depressive symptoms were assessed using the Calgary Depression Scale for Schizophrenia (CDSS) [43]. Medication side effects were evaluated by the Udvalg for Kliniske Undersøgelser (UKU) [44]. Medication adherence was assessed by a modified Cantonese version of the Medication Adherence Rating Scale [35, 45]. The type and frequency of the clinical care received over the four years were also recorded.

Functioning was indicated using the Social Occupational Functioning Assessment Scale (SOFAS) [46], the Role Functioning Scale (RFS) [47], and employment status (employed or not). Health-related quality of life was assessed by the 12-item Short-Form Health Survey (SF-12), which may be divided into physical and mental components [48].

Neurocognition was measured using a comprehensive battery of tests: information, arithmetic, digit span (forward and backward), digit symbol, logical memory test (total number of correct immediate and delayed recalls), semantic fluency test (total number of correct animals reported in one minute), visual patterns test (total number of correct responses), and the modified Wisconsin card sorting test (total number of perseveration errors).

### Statistical analysis

Statistical analyses were conducted using IBM^®^ SPSS^®^ version 28.0.1.0. First, baseline sociodemographic, premorbid, and help-seeking variables were compared between financially deprived and non-deprived groups using independent t-tests, Mann-Whitney U tests, or chi-squared tests, as appropriate.

Second, univariate linear and binary logistic regression analyses were performed to examine associations between baseline financial deprivation and baseline clinical, functional, and neurocognitive measures, adjusting for any significant sociodemographic, premorbid, or help-seeking covariates identified in the first step.

Third, the predictive value of baseline financial deprivation for 4-year clinical, functional, and neurocognitive outcomes was assessed using univariate linear and binary logistic regressions, controlling for baseline differences in DUP, schizophrenia diagnosis, place of birth, years of education, type of housing, and the corresponding baseline outcome measure. Covariates were identified based on baseline group comparisons (Table 1) and previous research demonstrating their impact on clinical and functional outcomes in psychosis [49–51]. The Benjamini-Hochberg procedure was applied to adjust for multiple comparisons (*q* = 0.10).

**Table 1:** Comparison of basic demographics, socioeconomic, premorbid, and help-seeking variables between financially deprived (*n* = 121) and financially non-deprived (*n* = 113) participants at baseline

	Deprived (*n* = 121)	Non-deprived (*n* = 113)	Χ_2_ / t / Z statistic (df)	*p*
*Basic demographics*				
Age at service entry, mean (SD)	38.65 (7.86)	37.13 (8.65)	*t*(232) = -1.41	0.160
Gender, *n* (%)				
Male	51 (42.1%)	55 (48.7%)	*X*_*2*_(1) = 1.00	0.316
Female	70 (57.9%)	58 (51.3%)		
**Years of education**,** mean (SD)**	**9.88 (3.53)**	**11.76 (3.79)**	***t*** **(232) = -3.92**	< 0.001**
Marital status, *n* (%)				
Married	39 (32.2%)	39 (34.5%)	*X*_*2*_(1) = 0.14	0.711
Others^1^	82 (67.8%)	74 (65.5%)		
Years of intervention, *n* (%)				
2 years	58 (47.9%)	59 (52.2%)	*X*_*2*_(1) = 0.43	0.513
4 years	63 (52.1%)	54 (47.8%)		
Family history of psychiatric disorders, *n* (%)	37 (30.6%)	28 (33.6%)	*X*_*2*_(1) = 0.25	0.617
Household size (including patient), mean (SD)	3.17 (1.62)	3.35 (1.38)	*t*(232) = -0.96	0.339
**Sibling size (including patient)**,** mean (SD)**	**4.22 (1.99)**	**3.55 (1.98)**	***t*** **(232) = 2.60**	0.010*
Living alone, *n* (%)	19 (15.7%)	10 (8.8%)	*X*_*2*_(1) = 2.53	0.112
**Smoker**, ***n*** **(%)**	**32 (26.4%)**	**16 (14.2%)**	***X***_***2***_ **(1) = 5.41**	0.020**
**Born in Hong Kong**, ***n*** **(%)**				
Yes	75 (62%)	89 (79%)	***X***_***2***_ **(1) = 7.85**	0.005**
No^2^	46 (38%)	24 (21%)		
*Socioeconomic variables*				
**Type of housing**, ***n*** **(%)**			***X***_***2***_ **(4) = 17.30**	0.004**
Public housing	82 (67.8%)	56 (49.6%)		
Subsidized sales flat	3 (2.5%)	17 (15.0%)		
Private permanent housing	32 (26.4%)	36 (31.9%)		
Temporary housing	3 (2.5%)	2 (1.8%)		
Non-domestic housing	0 (0%)	1 (0.9%)		
Others	1 (0.8%)	1 (0.9%)		
**Monthly household income**, ***n*** **(%)**				
HKD0-7,999	101 (83.5%)	60 (53.1%)	***X***_***2***_ **(1) = 98.29**	< 0.001**
HKD8000 or above	20 (16.5%)	53 (46.9%)		
**Monthly personal income**, ***n*** **(%)**				
HKD0-7,999	84 (69.4%)	7 (6.2%)	***X***_***2***_ **(1) = 25.12**	< 0.001**
HKD8000 or above	37 (30.6%)	106 (93.8%)		
*Premorbid characteristics*				
PAS overall, mean (SD)	0.20 (0.19)	0.16 (0.14)	*t*(231) = 1.88	0.062
PSST overall, mean (SD)	1.15 (0.26)	1.14 (0.20)	*t*(231) = 0.52	0.605
**DUP**,** days**,** median (IQR)**	119.00 (32.00–646.50)	63.00 (19.00–352.50)	***Z =*** **743.00**	0.047*
DI, days, median (IQR)	689.75 (143.00-758.50)	589.39 (118.00-444.00)	*Z =* 6031.00	0.120
Mode of onset, *n* (%)				
Acute (≤ 1 week)	27 (22.3%)	24 (21.2%)	*X*_*2*_(1) = 0.19	0.911
Sub-acute (1–4 weeks)	22 (18.2%)	23 (20.4%)		
Insidious (> 1 month)	72 (59.5%)	66 (58.4%)		
Number of stressful life events six months prior, mean (SD)	0.98 (1.30)	0.88 (1.27)	*t*(232) = 0.54	0.592
*Help-seeking characteristics*				
Total no. of help seeking, mean (SD)	2.46 (0.94)	2.43 (1.03)	*t*(232) = 0.23	0.821
Overall help seeking duration, mean (SD)	99.70 (245.37)	82.08 (165.17)	*t*(213) = 0.61	0.543
Help seeking delay, mean (SD)	569.26 (1093.87)	535.89 (1570.32)	*t*(215) = 0.18	0.855
System delay, mean (SD)	141.45 (247.85)	143.52 (174.01)	*t*(215) = -0.07	0.944

*Notes*. * *p* < 0.050, ** *p* < 0.010. Significant after the false discovery rate is calculated. All assessments were administered by trained research assistants who underwent intensive training in the use of study instruments

*Abbreviations.* SD - standard deviation, PAS - Pre-morbid Adjustment Scale, PSST - Assessment of Pre-morbid Schizoid-Schizotypal Traits, DUP - duration of untreated psychosis, IQR - interquartile range

^1^ Individuals who are single, divorced, widowed, or separated are defined as not married

^2^ Among all foreign-born, 98.6% (*n* = 69) were born in China and 1.4% (*n* = 1) were born in another country

Fourth, subgroup analyses were conducted to explore whether financial deprivation similarly predicts clinical, functional, and neurocognitive outcomes across age groups. Participants were divided into three age-based subgroups based on age at baseline, using tertiles (33rd and 66th percentiles) of the sample’s age distribution. The regression analyses described in the third step were repeated within each subgroup.

## Results

### Descriptive information

Of the 240 individuals selected for analyses, 234 provided complete household income data at baseline (Fig. 1).

### Baseline comparisons between financially deprived and non-deprived groups

#### Sociodemographic, premorbid, and help-seeking variables

Table 1 presents baseline comparisons across sociodemographic, premorbid, and help-seeking measures. Financially deprived participants had fewer years of education (*p* < 0.001), more siblings (*p* = 0.010), and were more likely to be smokers (*p* = 0.020) and foreign-born (*p* = 0.005). They were more frequently residents of public housing (*p* = 0.005) and less often in subsidized sale flats (*p* < 0.001). Compared with the non-deprived group, a larger proportion of deprived participants reported monthly household and personal incomes below HKD 8,000 (both *p* < 0.001). Deprived participants also had marginally longer DUP (*p* = 0.047), though this difference was not significant after false discovery rate correction.

#### Clinical, functional, and neurocognitive measures

Table 2 presents baseline comparisons of clinical, functional, and neurocognitive measures, adjusted for significant sociodemographic covariates identified in previous section. Financially deprived participants had a slightly lower proportion diagnosed with brief psychosis (*p* = 0.049). They also reported greater symptom severity, including higher total PANSS scores (*p* = 0.039), more severe negative symptoms (*p* = 0.036), and elevated avolition-apathy on the SANS (*p* = 0.040). This group also exhibited poorer functioning, with higher unemployment rates (*p* = 0.022) and lower RFS work productivity scores (*p* = 0.017). The two groups showed no statistically significant difference in their neurocognitive functions.

**Table 2:** Comparison of clinical, functional, and neurocognitive characteristics between financially deprived and financially non-deprived participants at baseline. ^1^

	Deprived (*n* = 121)	Non-deprived (*n* = 113)	B / OR	*p*	95% CI
*Demographics and diagnosis*					
Age at onset of psychosis, years, mean (SD)	36.79 (8.26)	35.55 (8.87)	1.939	0.112	[-0.458, 4.336]
Hospitalization at onset, *n* (%)	63 (52.1%)	65 (57.5%)	1.727	0.429	[0.446, 6.679]
Existing medical illness, *n* (%)	26 (21.5%)	21 (18.6%)	0.430	0.234	[0.107, 1.726]
Diagnosis at baseline, *n* (%)					
Schizophrenia	72 (59.5%)	53 (46.9%)	0.495	0.291	[0.135, 1.823]
** Brief psychosis**	**16 (13.2%)**	**24 (21.2%)**	**9.531**	0.049*	**[1.008**,** 90.083]**
Psychosis not otherwise specified	9 (7.4%)	10 (8.8%)	0.213	0.251	[0.015, 2.984]
Delusional disorder	20 (16.5%)	21 (18.6%)	1.695	0.506	[0.358, 8.020]
Bipolar with psychotic symptoms	1 (0.8%)	2 (1.8%)	3.614	0.590	[0.034, 386.136]
Schizoaffective disorder	1 (0.8%)	1 (0.9%)	0	0.865	0
Schizophreniform psychosis	2 (1.7%)	2 (1.8%)	-1.206	0.498	[0.009, 9.838]
*Clinical outcomes*,* mean (SD)*					
PANSS					
** Total**	**43.41 (12.07)**	**40.00 (10.54)**	**-7.017**	0.039*	**[-13.683**,** -0.350]**
Positive symptoms	9.39 (3.76)	8.81 (3.43)	-1.150	0.341	[-3.547, 1.247]
** Negative symptoms**	**10.47 (4.42)**	**9.68 (3.92)**	**-2.195**	0.036*	**[-4.239**,** -0.151]**
General psychopathology	23.55 (7.01)	21.50 (6.45)	-3.672	0.075	[-7.727, 0.384]
SAPS					
Total	5.45 (8.55)	4.32 (9.18)	-2.176	0.391	[-7.215, 2.863]
Hallucination	2.05 (4.06)	1.53 (4.04)	-0.995	0.441	[-3.561, 1.571]
Delusion	2.14 (3.82)	1.87 (4.18)	-0.594	0.641	[-3.126, 1.938]
Bizarre behavior	0.53 (1.69)	0.23 (1.13)	-0.442	0.351	[-1.383, 0.498]
Formal thought disorder	0.69 (2.38)	0.60 (2.90)	-0.145	0.784	[-1.198, 0.908]
Inappropriate affect	0.03 (0.26)	0.09 (0.47)	0.011	0.327	[-0.067, 0.199]
SANS					
Total	10.57 (13.60)	8.49 (13.30)	-5.633	0.103	[-12.435, 1.168]
Affective flattening	3.04 (5.86)	3.04 (5.83)	-0.692	0.646	[-3.696, 2.311]
Alogia	0.64 (2.06)	0.76 (2.21)	-0.234	0.672	[-1.332, 0.864]
** Avolition-apathy**	**2.16 (3.56)**	**1.33 (2.94)**	**-1.957**	0.040*	**[-3.822**,** -0.092]**
Anhedonia-asociality	4.30 (5.47)	2.96 (4.66)	-2.647	0.100	[-5.816, 0.522]
Attention	0.44 (1.62)	0.40 (1.52)	-0.103	0.630	[-0.528, 0.322]
Antipsychotic medication, *n* (%)			-1.130	0.234	[0.050, 2.074]
On medication	115 (95.0%)	110 (97.3%)			
Off medication	6 (5.0%)	3 (2.7%)			
Medication type, *n* (%)					
Conventional	35 (30.4%)	24 (21.8%)	2.135	0.352	[0.095, 756.302]
Atypical	79 (68.7%)	84 (76.4%)	1.908	0.410	[0.072, 629.402]
Both	1 (0.9%)	2 (1.8%)			
Antipsychotic medication chlorpromazine equivalent dosage	174.79 (159.37)	170.18 (151.90)	11.192	0.612	[-32.245, 54.630]
MCQ					
Attitude	3.16 (0.52)	3.25 (0.52)	0.130	0.364	[-0.155, 0.415]
Behavior	3.47 (0.59)	3.57 (0.53)	0.104	0.523	[-0.221, 0.430]
*Functional outcomes*,* mean (SD)*					
**Occupational status**, ***n*** **(%)**			**8.648**	0.022*	**[1.363**,** 54.855]**
Employed	45 (37.2%)	85 (75.2%)			
Unemployed	76 (62.8%)	28 (24.8%)			
Social and occupational functioning, mean (SD)					
SOFAS	51.91 (12.69)	57.81 (11.85)	6.005	0.099	[-1.156, 13.167]
** RFS work productivity**	**3.85 (1.44)**	**5.12 (1.36)**	**1.002**	0.017*	**[0.182**,** 1.823]**
RFS independent living, self-care	6.02 (1.06)	6.08 (1.05)	0.025	0.952	[-0.783, 0.833]
RFS immediate social network relationships	4.86 (1.39)	5.54 (1.14)	0.446	0.286	[-0.383, 1.274]
RFS extended social network relationships	4.12 (1.54)	4.71 (1.22)	0.546	0.238	[-0.370, 1.462]
*Neurocognitive outcomes*,* mean (SD)*					
Information (age adjusted)	8.01 (3.03)	9.28 (3.04)	-0.775	0.386	[-2.553, 1.003]
Arithmetic (age adjusted)	7.89 (2.99)	9.19 (2.68)	-0.439	0.575	[-1.996, 1.118]
Visual patterns test, correct items	14.89 (5.66)	16.54 (5.63)	-1.001	0.394	[-3.331, 1.329]
Digit symbol (age adjusted)	7.06 (2.67)	8.50 (3.17)	1.246	0.066	[-0.083, 2.576]
Digit span, forward	11.05 (2.50)	11.51 (2.38)	-0.750	0.406	[-2.541, 1.041]
Digit span, backward	5.79 (2.43)	6.82 (3.10)	0.119	0.877	[-1.409, 1.646]
Logical memory, immediate recall	8.33 (4.49)	9.59 (4.65)	-1.176	0.326	[-3.551, 1.199]
Logical memory, delayed recall	6.34 (4.60)	7.29 (4.80)	-0.562	0.655	[-3.071, 1.946]
Semantic fluency, correct response	14.87 (5.12)	17.04 (5.87)	-0.730	0.633	[-3.771, 2.311]

*Notes*. * *p* < 0.050, ** *p* < 0.010. Significant after the false discovery rate is calculated. All assessments were administered by trained research assistants who underwent intensive training in the use of study instruments

*Abbreviations.* PANSS - Positive and Negative Syndrome Scale, SAPS - Scale for Assessment of Positive Symptoms, SANS - Scale for Assessment of Negative Symptoms, MCQ - Medication Compliance Questionnaire, SOFAS - Social Occupational Functioning Assessment Scale, RFS - Role Functioning Scale

^1^ Univariate linear regressions/logistic regressions were conducted with financial deprivation status (deprived vs. not deprived) and seven potential confounding variables (DUP, years of education, place of birth, sibling size, smoking, type of housing, household income) as independent variables. Clinical, functional, and neurocognitive outcomes at 4 years were included as dependent variables. All assessments were administered by trained research assistants who underwent intensive training in the use of study instruments

### Predictive value of financial deprivation

#### Clinical, functional, and neurocognitive outcomes at four years

Baseline financial deprivation predicted worse clinical and functional outcomes at four-year follow-up (Table 3). Specifically, it was associated with higher PANSS scores for total symptoms (*p* = 0.004), positive symptoms (*p* = 0.035), negative symptoms (*p* = 0.018), and general psychopathology (*p* = 0.044). Similarly, financially deprived participants had higher SANS total scores (*p* < 0.001), as well as greater affective flattening (*p* < 0.001) and avolition-apathy (*p* < 0.001).

**Table 3:** Univariate linear and logistic regressions on whether financial deprivation at baseline predicts clinical, functioning, and neurocognitive outcomes at 4 years.^1^

	Deprived (*n* = 112)	Non-deprived (*n* = 107)	B / OR	*p*	95% CI
*Clinical outcomes at 4 years, mean (SD)*					
**PANSS**					
** Total**	**41.91 (8.13)**	**37.97 (5.65)**	**-2.79**	0.004**	**[-4.68**,** -0.90]**
** Positive symptoms**	**8.49 (2.68)**	**7.73 (1.97)**	**-0.69**	0.035*	**[-1.33**,** -0.05]**
** Negative symptoms**	**10.18 (3.81)**	**8.60 (2.22)**	**-1.04**	0.018*	**[-1.89**,** -0.18]**
** General psychopathology**	**20.15 (4.17)**	**18.53 (3.08)**	**-1.02**	0.044*	**[-2.02**,** -0.03]**
SAPS					
Total	2.71 (4.959)	1.51 (4.124)	-0.70	0.269	[-1.941, 0.544]
Hallucination	0.98 (2.585)	0.49 (1.824)	-0.41	0.202	[-1.042, 0.221]
Delusions	1.25 (3.458)	0.64 (2.089)	-0.39	0.334	[-1.176, 0.402]
Bizarre behavior	0.07 (0.532)	0.12 (0.669)	0.11	0.191	[-0.057, 0.283]
Formal thought disorder	0.37 (1.280)	0.27 (1.652)	0.04	0.866	[-0.375, 0.446]
Inappropriate affect	0.04 (0.266)	0.00 (0.000)	-0.04	0.175	[-0.095, 0.016]
SANS					
**Total**	**10.54 (11.78)**	**4.92 (6.30)**	**-4.70**	< 0.001**	**[-7.345**,** -2.056]**
** Affective flattening**	**2.57 (4.45)**	**0.93 (2.21)**	**-1.63**	< 0.001**	**[-2.585**,** -0.669]**
Alogia	0.59 (2.103)	0.33 (1.219)	0.000	0.999	[-0.481, 0.482]
** Avolition-apathy**	**2.79 (4.19)**	**1.06 (2.27)**	**-1.63**	< 0.001**	**[-2.589**,** -0.670]**
Anhedonia-asociality	4.26 (5.21)	2.50 (4.08)	-1.32	0.051	[-2.647, -0.003]
Attention	0.32 (1.28)	0.10 (0.75)	-0.09	0.546	[-0.381, 0.202]
CDSS	1.05 (2.00)	0.64 (1.35)	-0.14	0.558	[-0.599, 0.324]
Number of relapses in 4 years	0.71 (1.19)	0.67 (1.18)	-0.07	0.645	[-0.367, 0.228]
Ever relapsed in 4 years, n (%)	43 (36.8%)	38 (34.5%)	-0.14	0.640	[0.491, 1.549]
MCQ					
Behavior	3.48 (0.57)	3.52 (0.60)	0.07	0.428	[-0.107, 0.252]
Attitude	2.39 (0.41)	2.46 (0.43)	0.02	0.776	[-0.106, 0.142]
UKU					
Psychic	0.16 (0.24)	0.09 (0.18)	-0.04	0.284	[-0.099, 0.029]
Neurologic	0.04 (0.08)	0.02 (0.06)	-0.02	0.162	[-0.037, 0.006]
Autonomic	0.02 (0.06)	0.02 (0.07)	-0.00	0.729	[-0.023, 0.016]
Others	0.02 (0.04)	0.03 (0.06)	0.01	0.100	[-0.002, 0.027]
*Functional outcomes at 4 years*,* mean (SD)*					
**Unemployed**,** n (%)**	**40 (65.6%)**	**21 (34.4%)**	**0.83**	0.015*	**[1.173**,** 4.492]**
Social and occupational functioning					
** SOFAS**	**58.58 (10.17)**	**65.93 (8.19)**	**4.87**	< 0.001**	**[2.474**,** 7.263]**
RFS work productivity	4.77 (1.52)	5.55 (1.37)	0.32	0.133	[-0.098, 0.742]
RFS independent living, self-care	5.93 (0.79)	6.02 (0.88)	0.13	0.262	[-0.100, 0.366]
RFS immediate social network relationships	5.16 (1.02)	5.59 (0.97)	0.22	0.123	[-0.059, 0.489]
** RFS extended social network relationships**	**4.63 (1.04)**	**5.18 (1.04)**	**0.36**	0.013**	**[0.078**,** 0.646]**
Quality of life at 4 years					
** SF-12 physical component**	**65.34 (25.83)**	**77.31 (17.97)**	**10.83**	0.002**	**[4.199**,** 17.466]**
** SF-12 mental component**	**63.43 (24.86)**	**72.45 (20.26)**	**7.00**	0.043*	**[0.236**,** 13.760]**
*Neurocognitive outcomes at 4 years*,* mean (SD)*					
Visual patterns test, correct items	16.46 (6.278)	18.64 (6.071)	0.79	0.252	[-0.565, 2.144]
Semantic fluency, correct response	16.23 (6.07)	18.75 (6.48)	-0.01	0.990	[-1.482, 1.462]
Logical memory, immediate recall	9.76 (4.46)	10.58 (4.36)	-0.44	0.460	[-1.594, 0.724]
Logical memory, delayed recall	7.05 (4.25)	8.37 (5.01)	0.12	0.841	[-1.070, 1.313]
Digit symbol (age-adjusted)	8.48 (3.66)	9.89 (3.54)	-0.42	0.192	[-1.051, 0.212]
Digit span forward	11.91 (2.26)	12.42 (2.05)	0.19	0.443	[-0.300, 0.684]
Digit span backward	6.20 (2.90)	7.26 (3.29)	0.10	0.759	[-0.556, 0.762]
MWCST perseverative error	5.16 (5.82)	4.81 (6.48)	1.21	0.155	[-0.461, 2.883]

*Notes*. * *p* < 0.050, ** *p* < 0.010

*Abbreviations*. PANSS - Positive and Negative Syndrome Scale, SAPS - Scale for Assessment of Positive Symptoms, SANS - Scale for Assessment of Negative Symptoms, CDSS - Calgary Depression Scale for Schizophrenia, MCQ - Medication Compliance Questionnaire, UKU - Udvalg for Kliniske Undersøgelser, SOFAS - Social and Occupational Functioning Scale, RFS - Role Functioning Scale, SF-12–12-item Short-Form Survey, MWCST - Modified Wisconsin Card Sorting Test

^1^ Univariate linear regressions/logistic regressions were conducted with financial deprivation status (deprived vs. not deprived) and five potential confounding variables (DUP, years of education, schizophrenia diagnosis, place of birth, type of housing), and the baseline score of the variable as independent variables. Clinical, functional, and neurocognitive outcomes at 4 years were included as dependent variables. All assessments were administered by trained research assistants who underwent intensive training in the use of study instruments

Financial deprivation also predicted poorer functional outcomes, including a lower likelihood of being employed (*p* = 0.015), lower SOFAS scores (*p* < 0.001), and smaller extended social networks on the RFS (*p* = 0.013). In addition, participants experiencing financial deprivation at baseline reported worse self-perceived quality of life on the SF-12, both physically (*p* = 0.002) and mentally (*p* = 0.043) at follow-up.

Financially deprived status at baseline did not significantly predict neurocognitive performance at four years.

#### Public and private clinical care received over 4 years

More financially deprived participants used public healthcare services over the four-year follow-up, including specialist outpatient clinics (*p* = 0.020) and community psychiatric nurses (*p* = 0.021). Further, fewer of them visited private general practitioners (*p* = 0.006) relative to the non-deprived group (Table 4**)**.

**Table 4:** Public and private clinical care received over 4 years in the deprived and non-deprived participants.^1^

	Deprived (*n* = 121)	Non-deprived (*n* = 113)	t statistic (df)	*p*
*Type of public healthcare, mean (SD)*				
General outpatient clinic	0.19 (0.59)	0.12 (0.35)	*t*(208) = 1.14	0.256
** Specialist outpatient clinic**	**5.25 (1.55)**	**4.77 (1.37)**	***t*** **(208) = 2.35**	0.020*
Accident and Emergency	0.06 (0.23)	0.04 (0.20)	*t*(208) = 0.55	0.581
Chinese medicine	0.06 (0.36)	0.01 (0.10)	*t*(208) = 1.25	0.213
Medical social worker	0.22 (0.50)	0.33 (1.30)	*t*(206) = -0.80	0.425
** Community psychiatric nurse**	**0.46 (0.80)**	**0.22 (0.69)**	***t*** **(208) = 2.33**	0.021*
Case manager	0.81 (0.94)	0.77 (1.07)	*t*(207) = 0.24	0811
Occupational therapist	0.19 (1.05)	0.20 (0.91)	*t*(207) = -0.03	0.979
Physiotherapist	0.04 (0.27)	0.00 (0.00)	*t*(207) = 1.37	0.171
Clinical psychologist	0.05 (0.25)	0.03 (0.22)	*t*(207) = 0.51	0.614
Dietitian	0.01 (0.10)	0.00 (0.00)	*t*(207) = 0.97	0.335
Social worker	0.42 (1.60)	0.19 (0.70)	*t*(207) = 1.32	0.188
Day training	0.69 (4.09)	0.18 (1.29)	*t*(207) = 1.19	0.235
Supported employment	0.17 (1.16)	0.06 (0.37)	*t*(207) = 0.89	0.377
*Type of private healthcare*,* mean (SD)*				
** General practitioner**	**0.14 (0.48)**	**0.45 (1.05)**	***t*** **(207) = -2.77**	0.006**
Specialist	0.06 (0.27)	0.29 (2.00)	*t*(207) = -1.22	0.225
Psychiatrist	0.03 (0.17)	0.10 (0.39)	*t*(207) = -1.72	0.087
Chinese medicine	0.25 (1.56)	0.29 (1.87)	*t*(207) = -0.72	0.861

*Notes*. * *p* < 0.050, ** *p* < 0.010

^1^ Number of visits to healthcare professionals within two weeks prior to the assessment time points (at baseline, 1-year, 2-year, 3-year, 4-year follow-up) was measured. The amount of clinical care received was the sum of visits recorded over the four-year period

### Effect of financial deprivation across age

Participants were divided into three subgroups based on age at baseline: <33 years (*n* = 75), 33–42 years (*n* = 94), and > 42 years (*n* = 71). Detailed results are provided in **Online Resource 2**. In the < 33 years subgroup, baseline financial deprivation did not predict any 4-year outcomes (*p* > 0.05).

In the 33–42 years subgroup, financial deprivation was associated with fewer UKU ‘other’ side effects (*p* = 0.037) and poorer self-reported physical health on the SF-12 (*p* = 0.009) at four years.

The strongest and most consistent associations were observed in the > 42 years subgroup. Baseline financial deprivation predicted worse 4-year clinical outcomes, including higher PANSS total (*p* = 0.011), negative (*p* = 0.019), and general psychopathology scores (*p* = 0.028), as well as higher SANS total (*p* = 0.006), affective flattening (*p* = 0.021), avolition-apathy (*p* = 0.043), and anhedonia-asociality (*p* = 0.007). Functioning was also poorer, with lower SOFAS (*p* < 0.001), as well as RFS work productivity (*p* = 0.029), and independent living scores (*p* = 0.012). Further, financially deprived participants reported worse physical (*p* = 0.023) and mental health (*p* = 0.044) on the SF-12 at follow-up.

## Discussion

This study is among the first in Hong Kong to examine the prospective relationship between financial deprivation during early psychosis and long-term outcomes, with consideration of age-related differences. At baseline, financially deprived individuals with FEP were characterized by fewer years of education, more siblings, higher smoking rates, foreign-born status, and residence in public housing, reflecting a complex interplay between socioeconomic disadvantage and mental health. Importantly, financial deprivation predicted poorer symptomatic and functional outcomes at four years. Subgroup analyses further indicated that these effects were most pronounced in older, financially-deprived participants, who experienced greater symptom severity, worse functioning, and lower quality of life at four years. These findings highlight the importance of considering age when assessing the impact of financial deprivation and suggest that older, socioeconomically disadvantaged adults with psychosis may be particularly vulnerable, underscoring the need for age-sensitive clinical interventions and targeted policy measures.

### Financial deprivation in Hong Kong

Over half of our sample (51.7%) experienced financial deprivation during FEP, which is considerably higher than that of the general Hong Kong population in the corresponding year (15.2%) [29] and a comparable US-based study (30.1%) [52]. This discrepancy may partly reflect inconsistencies in how poverty is defined across studies. In the present study, financial deprivation was defined using the Hong Kong government’s official poverty line, which classifies households with income below 50% of the median (adjusted for household size) as living in poverty [29]. Indeed, this relative definition may make existing wealth disparities more evident, where many may still fall below the poverty line even if absolute incomes rise, creating the perception of high poverty rates in a wealthy city with a high cost of living. Hong Kong’s extreme wealth inequality (Gini coefficient = 0.54 in 2016) [21] may further elevate this threshold.

However, household income alone does not fully capture socioeconomic disadvantage. Other dimensions, including occupation, education [53, 54], neighborhood-level deprivation [52], material hardship [55], food insecurity [56], and subjective financial strain [57], also shape illness outcomes. For instance, research has shown that parental education levels are linked to children’s vulnerability to mental health issues under stress [58]. Others have also adopted more multifaceted definitions, operationalizing financial deprivation as household income falling below the minimum income needed to afford housing, food, and other basic living expenses [52]. While our study did not comprehensively assess all aspects of socioeconomic background, baseline comparisons showed that financially deprived participants faced broader disadvantages, such as lower education levels and worse housing conditions. These findings underscore the multidimensional and context-specific nature of financial deprivation in Hong Kong.

### Correlates of financial deprivation

Financially deprived individuals were characterized by fewer years of education, more severe general psychopathology, as well as poorer social and occupational functioning. They were also more likely to be foreign-born, primarily from Mainland China, with a smaller number from other regions. Local evidence suggests that Mainland Chinese immigrants in Hong Kong may face additional socioeconomic challenges, including generally lower educational attainment, reduced employment opportunities, concentration in low-skilled jobs, and local exclusionary attitudes towards their access to social assistance [59, 60]. Nevertheless, some immigrants may draw on family or community networks that buffer against financial strain [61].

It is also important to note that financial deprivation rarely occurs in isolation, instead intersecting with factors such as education, housing, employment, and demographics. Research in Hong Kong (*n* = 1,668) found that older age, female gender, lack of a partner, single-parent household, unemployment, residence in public rental housing, lower educational attainment, and poorer self-rated health were all associated with a greater risk of poverty [19]. Consistent with this, our baseline correlates analysis showed that financially deprived individuals shared several of these disadvantages. Prior research highlights that these overlapping influences often cluster and reinforce one another, creating cycles of disadvantage that are difficult to disentangle or address individually [18]. Taken together, these results highlight financial deprivation as a constellation of structural and individual disadvantages that intersect with social, cultural, and economic conditions to shape recovery trajectories in early psychosis.

### Financial deprivation as a predictor of 4-year outcomes

Our findings indicate that individuals experiencing financial deprivation reported greater psychosis symptom severity at the four-year follow-up. These findings align with prior research linking low socioeconomic status to more severe positive symptoms at six-month follow-up [5] and increased likelihood of persistent or residual symptoms at 14-year follow-up [8]. Financially deprived individuals were also more likely to rely on public healthcare services, such as specialist outpatient clinics and community nursing, and less likely to access private general practitioners. They also had longer DUP (four months vs. two months), which may reflect barriers within the city’s fragmented social safety net [22] and contribute to worse symptomatic outcomes [50]. Additional environmental stressors, including impoverished neighborhoods and substandard housing conditions, may further restrict access to social resources critical for recovery [62, 63].

Consistent with prior findings [4], functional impairments were also observed at the four-year mark, including worse overall functioning, weaker extended social relationships, lower employment rates, and reduced subjective physical and mental quality of life. These results suggest that economic strain extends beyond financial hardship, adversely impacting social integration, overall well-being, and access to care. While causality cannot be established, this study provides robust evidence that financial deprivation is associated with poor long-term outcomes in psychosis, highlighting the need to explore possible mechanisms such as the social drift hypothesis, where psychosis onset may precipitate downward social mobility [64].

### Age-specific effects of financial deprivation

Our findings indicate that the impact of financial deprivation on long-term outcomes in psychosis is age-dependent, with effects varying across developmental periods. Participants were divided into three age-based subgroups at baseline, representing distinct life stages with differing milestones, such as career establishment, family responsibilities, and age-related functional changes. The strongest and most consistent associations were observed in the later adulthood group (> 42 years), where financial deprivation predicted a wide range of poorer 4-year outcomes, including greater symptom burden, reduced functioning, and worse self-reported quality of life. In contrast, financial deprivation had minimal impact in early adulthood (< 33 years) and only modest effects in mid-adulthood (33–42 years), suggesting that the adverse effects of financial hardship may compound over time.

Several mechanisms may underlie these age-specific effects. Older adults with psychosis may face additional challenges in managing financial responsibilities in the context of cognitive and functional limitations. For example, qualitative evidence from healthcare professionals highlights the added challenges that older individuals encounter in navigating increasingly digitalized financial and healthcare systems [18]. In another study of adults aged 45 to 86 with schizophrenia-spectrum disorders, these individuals exhibited greater difficulties in everyday tasks compared with age-matched peers, including recalling items for shopping or completing routine activities, likely reflecting attentional and memory impairments on top of typical age-related declines [65].

Taken together, these findings suggest that financial hardship interacts with age-related cognitive and functional changes, particularly when psychosis onset occurs later in adulthood. This underscores the need for age-sensitive interventions that address both socioeconomic disadvantage and life-stage-specific challenges, including support for daily living, financial management, and social integration, to mitigate long-term functional decline.

### Limitations and future directions

This study has several limitations. First, its naturalistic observational design precludes causal inferences. The relationship between psychosis and financial deprivation is likely dynamic: socioeconomic disadvantage may increase vulnerability to illness or hinder recovery, while psychosis itself can precipitate downward social mobility. Other sociodemographic factors, such as education, housing, and immigration status, may also confound observed relationships. Although we adjusted for key covariates, residual confounding cannot be ruled out. Future longitudinal and qualitative studies are needed to better capture these bidirectional influences, disentangle confounding influences, and explore the lived experiences of financial hardship in psychosis.

Second, financial deprivation was assessed only at baseline. Household income and financial circumstances may fluctuate substantially over time, with some individuals moving into or out of financial deprivation during follow-up. Without capturing these shifts, the present study could not evaluate how changes in financial status shape outcomes. Future research should adopt longitudinal designs that track socioeconomic conditions at multiple time points. Such work would clarify whether improvements in financial circumstances buffer against poor illness outcomes, or whether worsening financial hardship compounds risk, thereby informing more responsive clinical interventions and social policies.

Third, financial deprivation was defined solely by monthly household income relative to the Hong Kong government’s official poverty line (< 50% of the median, adjusted for household size). This threshold may partly explain the high prevalence observed in our sample (51.7% vs. 15.2% in the general population). While it allows comparability with official statistics, it represents only one operationalization of deprivation. Further, in a context of extreme income inequality, relative deprivation, through processes such as social comparison [66], may exert greater psychosocial stress beyond absolute poverty alone, which may in turn impact illness trajectories. Nonetheless, household income alone does not fully capture the multidimensional or psychosocial aspects of disadvantage. Broader indicators, such as neighborhood-level deprivation, material hardship, food insecurity, and subjective financial strain, could provide a more comprehensive understanding of how socioeconomic factors shape illness trajectories. Future research would benefit from incorporating multiple indicators to better understand the impact of financial deprivation on individuals with psychosis and to inform more targeted care strategies.

Last, our participants were drawn exclusively from individuals enrolled in the JCEP project [28], which may limit generalizability to other early psychosis populations.

### Implications

The current findings carry important implications for clinical practice and mental health policy. Individuals with early psychosis who also experience financial deprivation reported more severe symptoms and impaired functioning at both illness onset and four years later, with the strongest and most consistent effects observed in those over 42 years. Clinicians should therefore consider service users’ financial circumstances during assessment and treatment planning, particularly for older adults who may be at greater risk of cumulative functional decline. Age-sensitive interventions, such as vocational support, assistance with accessing government subsidies or social benefits, and programs promoting social engagement and independent living skills, may help mitigate these risks. At a policy level, targeted support for socioeconomically disadvantaged groups, including measures to improve employment opportunities, facilitate access to healthcare, and address barriers faced by immigrant populations, is essential to buffer the long-term effects of financial deprivation on illness trajectories across the lifespan.

## Supplementary Information

Below is the link to the electronic supplementary material.

Supplementary Material 1

## Data Availability

The datasets used and/or analyzed during the current study are available from the corresponding author on reasonable request.
