# Supplementary material for: Impact of financial deprivation in first-episode psychosis: a prospective 4-year follow-up study
Source: Soc Psychiatry Psychiatr Epidemiol. 2025 Oct 17;61(2):283–96. doi: 10.1007/s00127-025-03006-y (PMC12948834; doi:10.1007/s00127-025-03006-y)
Supplement: Supplementary file 1 — Supplementary Material 1 [file 127_2025_3006_MOESM1_ESM.docx]

**Supplementary Materials**

Christy LMH, Eddie CYL, Charlie CLW, Impact of financial deprivation in first-episode psychosis: A prospective 4-year follow-up study.

**Supplementary material 1.** Poverty lines by household size according to 2012 Population Census in Hong Kong and the distribution of participants across different household sizes in deprived and non-deprived groups.

**Supplementary material 2.** Univariate linear and logistic regressions on whether financial deprivation at baseline predicts clinical, functioning, and neurocognitive outcomes at 4 years across age.

**References**

This supplemental material has been provided by the authors to give readers additional information about their work.

**Supplementary Material 1.** Poverty lines by household size according to the 2012 Population Census in Hong Kong, and the distribution of participants across different household sizes in deprived and non-deprived groups [1].

| Household size | Deprived (n=121) | Non-deprived (n=113) | Poverty line |
| --- | --- | --- | --- |
|  | *N* (%) | |  |
| One person | 19 (15.7%) | 10 (8.8%) | HK$3,600 |
| Two persons | 24 (19.8%) | 19 (16.8%) | HK$7,700 |
| Three persons | 27 (22.3%) | 34 (30.1%) | HK$11,500 |
| Four persons | 34 (28.1%) | 30 (26.5%) | HK$14,300 |
| Five persons | 11 (9.1%) | 14 (12.4%) | HK$14,800 |
| Six persons or more | 6 (5%) | 6 (5.3%) | HK$15,800 |

*Note.* In this study, the threshold for financial deprivation is half of the median household income recorded in the 2012 Population Census due to its proximity to the data collection period (Census and Statistics Department, 2012). The threshold values are determined separately for households of different sizes.

**Supplementary Material 2.** Univariate linear and logistic regressions on whether financial deprivation at baseline predicts clinical, functioning, and neurocognitive outcomes at 4 years across age

|  | Age group 1: <33 years old (n=75) | | | | | Age group 2: >=33 and <=42 years old (n=94) | | | | | Age group 3: >42 years old (n=71) | | | | |
| --- | --- | --- | --- | --- | --- | --- | --- | --- | --- | --- | --- | --- | --- | --- | --- |
| Continuous variables | Deprived group  (*n*=30) | Non-deprived group  (*n*=45) | *OR (ExpB)* | *p (Sig)* | 95% CI | Deprived group  (*n*=53) | Non-deprived group  (*n*=35) | *OR (ExpB)* | *p (Sig)* | 95% CI | Deprived group  (*n*=38) | Non-deprived group  (*n*=33) | *OR (ExpB)* | *p (Sig)* | 95% CI |
| *Clinical outcomes at 4 years, mean (SD)* | | | | | | | | | | | | | | | |
| PANSS |  |  |  |  |  |  |  |  |  |  |  |  |  |  |  |
| Total | 44.68 (9.83) | 38.31 (6.18) | -1.286 | 0.460 | [-4.744, 2.172] | 40.18 (6.46) | 36.88 (3.53) | -1.916 | 0.160 | [-4.604, 0.773] | **44.68 (9.83)** | **38.31 (6.18)** | **-5.673** | **0.011*** | **[-10.000, -1.346]** |
| Positive symptoms | 8.15 (1.89) | 7.41 (1.12) | -0.819 | 0.302 | [-2.393, 0.755] | 8.32 (2.65) | 7.47 (0.10) | -0.680 | 0.167 | [-1.651, 0.292] | 8.15 (1.89) | 7.41 (1.12) | -0.592 | 0.159 | [-1.424, 0.239] |
| Negative symptoms | 11.41 (4.52) | 8.79 (2.74) | 0.220 | 0.739 | [-1.093, 1.533] | 9.86 (3.47) | 8.18 (1.64) | -0.722 | 0.263 | [-1.997, 0.553] | **11.41 (4.50)** | **8.79 (2.74)** | **-2.472** | **0.019*** | **[-4.521, -0.424]** |
| General  psychopathology | 22.00 (5.17) | 18.90 (3.31) | -0.717 | 0.393 | [-2.382, 0.949] | 18.92 (3.10) | 18.15 (2.56) | -0.449 | 0.543 | [-1.912, 1.014] | **22.00 (5.17)** | **18.90 (3.31)** | **-2.533** | **0.028*** | **[-4.775, -0.290]** |
| SAPS |  |  |  |  |  |  |  |  |  |  |  |  |  |  |  |
| Total | 2.29 (2.82) | 1.07 (2.85) | -1.224 | 0.455 | [-4.475, 2.028] | 2.02 (4.35) | 0.97 (2.15) | -0.501 | 0.556 | [-2.191, 1.189] | 2.29 (2.82) | 1.07 (2.85) | -0.847 | 0.251 | [-2.309, 0.615] |
| Hallucination | 1.03 (2.10) | 0.59 (1.92) | -0.598 | 0.351 | [-1.869, 0.673] | 0.98 (2.89) | 0.18 (1.03) | -0.542 | 0.349 | [-1.689, 0.604] | 1.03 (2.10) | 0.59 (1.92) | -0.233 | 0.659 | [-1.284, 0.819] |
| Delusions | 0.59 (1.44) | 0.59 (1.92) | -0.918 | 0.384 | [-3.013, 1.177] | 0.68 (2.16) | 0.53 (1.66) | 0.121 | 0.796 | [-0.811, 1.054] | 0.59 (1.44) | 0.00 (0.00) | -0.450 | 0.115 | [-1.012, 0.112] |
| Bizarre behavior | 0.00 (0.00) | 0.32 (1.61) | 0.058 | 0.803 | [-0.404, 0.520] | 0.00 (0.00) | 0.12 (0.69) | 0.168 | 0.137 | [-0.054, 0.390] | 0.00 (0.00) | 0.07 (0.37) | 0.069 | 0.318 | [-0.068, 0.206] |
| Formal thought disorder | 0.68 (1.41) | 0.41 (2.23) | 0.055 | 0.875 | [-0.643, 0.754] | 0.32 (1.48) | 0.15 (0.50) | -0.231 | 0.417 | [-0.795, 0.333] | 0.68 (1.41) | 0.41 (2.23) | -0.272 | 0.572 | [-1.229, 0.686] |
| Inappropriate affect ^a^ | 0.00 (0.00) | 0.05 (0.30) | -0.039 | 0.445 | [-0.141, 0.063] | 0.04 (0.28) | 0.00 (0.00) | -0.055 | 0.324 | [-0.165, 0.055] | 0.00 (0.00) | 0.00 (0.00) | - | - | - |
| SANS |  |  |  |  |  |  |  |  |  |  |  |  |  |  |  |
| Total | 12.79 (12.50) | 4.10 (6.85) | -1.718 | 0.115 | [-3.868, 0.431] | 9.28 (9.71) | 5.62 (6.56) | -0.946 | 0.653 | [-5.115, 3.223] | **12.79 (12.50)** | **4.10 (6.85)** | **-7.787** | **0.006*** | **[-13.297, -2.278]** |
| **Affective flattening** | **2.74 (4.10)** | **0.79 (4.10)** | **-1.63** | **<0.001**** | **[-2.585, -0.669]** | 2.38 (3.71) | 0.88 (2.27) | -1.084 | 0.137 | [-2.520, 0.353] | **2.74 (4.10)** | **0.79 (4.10)** | **-2.088** | **0.021*** | **[-3.844, -0.332]** |
| Alogia | 1.15 (3.34) | 0.52 (1.62) | 0.077 | 0.807 | [-0.555, 0.710] | 0.38 (1.19) | 0.24 (0.96) | 0.026 | 0.922 | [-0.509, 0.562] | 1.15 (3.34) | 0.52 (1.62) | -0.465 | 0.508 | [-1.862, 0.933] |
| Avolition-apathy | 3.12 (5.09) | 0.55 (1.76) | -1.052 | 0.191 | [-2.644, 0.539] | 2.38 (3.56) | 1.12 (2.48) | -0.705 | 0.369 | [-2.257, 0.848] | **3.12 (5.09)** | **0.55 (1.76)** | **-2.157** | **0.043*** | **[-4.239, -0.076]** |
| Anhedonia-asociality | 5.47 (5.17) | 1.86 (3.06) | -1.159 | 0.350 | [-3.621, 1.303] | 3.72 (5.03) | 3.38 (4.93) | 0.773 | 0.540 | [-1.730, 3.276] | **5.47 (5.17)** | **1.86 (3.06)** | **-3.331** | **0.007*** | **[-5.706, -0.956]** |
| Attention | 0.38 (1.43) | 2.47 (4.17) | -0.178 | 0.142 | [-0.418, 0.061] | 0.42 (1.57) | 0.00 (0.00) | -0.137 | 0.638 | [-0.716, 0.442] | 0.32 (1.17) | 0.38 (1.43) | 0.179 | 0.604 | [-0.510, 0.869] |
| CDSS | 1.68 (2.73) | 0.76 (1.60) | -0.198 | 0.624 | [-0.999, 0.603] | 0.52 (1.02) | 0.42 (1.55) | -0.004 | 0.985 | [-0.482, 0.473] | 1.68 (2.73) | 0.76 (1.60) | 0.014 | 0.982 | [-1.246, 1.274] |
| Number of relapses in 4 years | 0.58 (1.00) | 0.52 (0.93) | -0.099 | 0.741 | [-0.695, 0.497] | 0.79 (1.43) | 0.74 (1.31) | -0.052 | 0.861 | [-0.646, 0.541] | 0.58 (1.00) | 0.52 (0.93) | -0.062 | 0.770 | [-0.484, 0.360] |
| MCQ |  |  |  |  |  |  |  |  |  |  |  |  |  |  |  |
| Behavior | 3.68 (0.45) | 3.62 (0.67) | -0.049 | 0.715 | [-0.316, 0.218] | 3.33 (0.70) | 3.59 (0.51) | 0.339 | 0.076 | [-0.037, 0.714] | 3.68 (0.45) | 3.62 (0.67) | -0.110 | 0.480 | [-0.420, 0.201] |
| Attitude | 2.41 (0.42) | 2.46 (0.40) | 0.152 | 0.177 | [-0.071, 0.375] | 2.44 (0.42) | 2.44 (0.51) | -0.116 | 0.327 | [-0.350, 0.119] | 2.41 (0.42) | 2.45 (0.40) | -0.089 | 0.430 | [-0.313, 0.135] |
| UKU |  |  |  |  |  |  |  |  |  |  |  |  |  |  |  |
| Psychic | 0.21 (0.28) | 0.15 (0.28) | -0.071 | 0.137 | [-0.166, 0.023] | 0.13 (0.21) | 0.10 (0.14) | -0.004 | 0.941 | [-0.103, 0.096] | 0.21 (0.28) | 0.15 (0.28) | -0.032 | 0.700 | [-0.197, 0.134] |
| Neurologic | 0.06 (0.08) | 0.03 (0.08) | 0.002 | 0.923 | [-0.033, 0.036] | 0.04 (0.09) | 0.02 (0.04) | -0.007 | 0.706 | [-0.046, 0.032] | 0.06 (0.08) | 0.03 (0.08) | -0.030 | 0.193 | [-0.076, 0.016] |
| Autonomic | 0.04 (0.08) | 0.03 (0.10) | 0.007 | 0.626 | [-0.023, 0.037] | 0.02 (0.05) | 0.01 (0.04) | -0.018 | 0.136 | [-0.042, 0.006] | 0.04 (0.08) | 0.03 (0.07) | -0.006 | 0.838 | [-0.060, 0.049] |
| Others | 0.02 (0.05) | 0.03 (0.07) | 0.012 | 0.359 | [-0.014, 0.037] | **0.02 (0.04)** | **0.03 (0.05)** | **0.025** | **0.037*** | **[0.002, 0.048]** | 0.02 (0.07) | 0.06 (0.17) | -0.003 | 0.841 | [-0.037, 0.030] |
| *Functional outcomes at 4 years, mean (SD)* | | | | | | | | | | | | | | | |
| SOFAS | 56.35 (9.53) | 66.93 (7.04) | 2.567 | 0.293 | [-2.269, 7.403] | 59.56 (10.02) | 66.18 (7.32) | 3.198 | 0.129 | [-0.947, 7.343] | **56.35 (9.53)** | **66.93 (7.04)** | **7.997** | **<0.001*** | **[3.858, 12.136]** |
| RFS work productivity | 22.97 (2.91) | 4.50 (1.69) | -0.158 | 0.692 | [-0.952, 0.636] | 4.86 (1.34) | 5.53 (1.48) | 0.061 | 0.869 | [-0.675, 0.798] | **4.05 (1.23)** | **5.24 (1.39)** | **0.819** | **0.029*** | **[0.085, 1.554]** |
| RFS independent living, self-care | 5.79 (1.10) | 6.38 (0.56) | -0.288 | 0.280 | [-0.816, 0.240] | 5.98 (0.69) | 6.09 (0.75) | 0.134 | 0.439 | [-0.209, 0.477] | **5.79 (0.91)** | **6.38 (0.56)** | **0.538** | **0.012*** | **[0.125, 0.952]** |
| RFS immediate social network relationships | 5.03 (1.03) | 5.52 (1.09) | 0.061 | 0.825 | [-0.487, 0.608] | 5.22 (1.00) | 5.76 (0.70) | 0.388 | 0.091 | [-0.063, 0.838] | 5.03 (1.03) | 5.52 (1.09) | 0.240 | 0.371 | [-0.293, 0.772] |
| Continuous variables | Deprived group  (*n*=30) | Non-deprived group  (*n*=45) | *OR (ExpB)* | *p (Sig)* | 95% CI | Deprived group  (*n*=53) | Non-deprived group  (*n*=35) | *OR (ExpB)* | *p (Sig)* | 95% CI | Deprived group  (*n*=38) | Non-deprived group  (*n*=33) | *OR (ExpB)* | *p (Sig)* | 95% CI |
| RFS extended social network relationships | 4.47 (1.08) | 5.14 (1.16) | 0.217 | 0.461 | [-0.368, 0.802] | 4.68 (1.00) | 5.32 (0.73) | 0.415 | 0.053 | [-0.006, 0.836] | 4.47 (1.08) | 5.14 (1.16) | 0.493 | 0.099 | [-0.096, 1.082] |
| *Quality of life at 4 years, mean (SD)* | |  |  |  |  |  |  |  |  |  |  |  |  |  |  |
| SF-12 physical component | 53.29 (23.95) | 65.56 (30.95) | -1.931 | 0.731 | [-13.136, 9.274] | **71.37 (21.40)** | **75.74 (22.04)** | **13.673** | **0.009*** | **[3.579, 23.766]** | **53.56 (23.95)** | **65.56 (30.95)** | **17.362** | **0.023*** | **[2.497, 32.227]** |
| SF-12 mental component | 49.52 (27.63) | 65.39 (28.53) | 1.326 | 0.827 | [-10.800, 13.452] | **61.10 (24.36)** | **66.50 (23.04)** | **5.675** | **0.340** | **[-6.146, 17.496]** | **49.52 (27.63)** | **65.39 (28.53)** | **13.105** | **0.044*** | **[0.338, 25.873]** |
| *Neurocognitive outcomes at 4 years, mean (SD)* | | | | | | | | | | | | | | | |
| Visual patterns test, correct items | 12.97 (5.21) | 13.75 (4.91) | 0.540 | 0.667 | [-1.963, 3.044] | 17.24 (6.24) | 19.33 (5.48) | -0.621 | 0.627 | [-3.162, 1.921] | 12.97 (5.21) | 13.75 (4.91) | 1.541 | 0.164 | [-0.653, 3.736] |
| Semantic fluency, correct response | 15.63 (6.15) | 14.71 (5.41) | 1.056 | 0.455 | [-1.764, 3.875] | 15.91 (5.75) | 19.73 (6.71) | -1.565 | 0.302 | [-4.579, 1.449] | 15.63 (6.15) | 14.71 (5.41) | -0.247 | 0.854 | [-2.937, 2.443] |
| Logical memory, immediate recall | 9.34 (4.63) | 9.54 (4.14) | -0.384 | 0.732 | [-2.619, 1.851] | 10.00 (4.01) | 11.07 (4.32) | -0.179 | 0.865 | [-2.271, 1.913] | 9.34 (4.63) | 9.54 (4.14) | 0.167 | 0.882 | [-2.075, 2.408] |
| Logical memory, delayed recall | 6.22 (4.21) | 7.33 (4.34) | -1.124 | 0.361 | [-3.571, 1.323] | 7.03 (4.00) | 9.00 (4.87) | 1.053 | 0.327 | [-1.078, 3.183] | 6.22 (4.21) | 7.33 (4.34) | 0.512 | 0.604 | [-1.458, 2.482] |
| Digit symbol (age-adjusted) | 7.03 (3.47) | 7.92 (3.44) | -0.805 | 0.172 | [-1.971, 0.362] | 8.55 (3.62) | 10.07 (3.25) | -0.665 | 0.256 | [-1.827, 0.496] | 7.03 (3.47) | 7.92 (3.44) | 0.130 | 0.836 | [-1.122, 1.381] |
| Digit span forward | 11.27 (2.81) | 11.08 (3.02) | 0.633 | 0.070 | [-0.052, 1.319] | 12.38 (1.98) | 12.87 (1.50) | -0.065 | 0.874 | [-0.886, 0.755] | 11.27 (2.81) | 11.08 (3.02) | 0.048 | 0.932 | [-1.073, 1.168] |
| Digit span backward | 5.03 (2.83) | 5.79 (2.96) | -0.078 | 0.891 | [-1.207, 1.051] | 6.44 (2.77) | 7.63 (3.55) | -0.350 | 0.572 | [-1.582, 0.881] | 5.03 (2.83) | 5.79 (2.86) | 0.754 | 0.217 | [-0.456, 1.965] |
| MWCST perseverative error | 6.74 (6.56) | 8.63 (9.01) | 0.967 | 0.239 | [-0.664, 2.597] | 5.92 (6.05) | 4.60 (5.51) | 1.982 | 0.159 | [-0.799, 4.763] | 6.74 (6.56) | 8.63 (9.01) | 2.387 | 0.262 | [-1.842, 6.616] |
| Categorical variables | Deprived group  (*n* = 30) | Non-deprived group  (*n* = 45) | *OR (ExpB)* | *P (Sig)* | 95% CI | Deprived group  (*n*=53) | Non-deprived group  (*n*=35) | *OR (ExpB)* | *p (Sig)* | 95% CI | Deprived group  (*n*=38) | Non-deprived group  (*n*=33) | *OR (ExpB)* | *p (Sig)* | 95% CI |
| Ever relapsed in 4 years, n (%) | 13 (41.9%) | 18 (58.1%) | 0.711 | 0.531 | [0.245, 2.067] | 31 (58.5%) | 22 (64.7%) | 0.948 | 0.916 | [0.347, 2.586] | 18 (47.4%) | 31 (93.9%) | 1.195 | 0.767 | [0.369, 3.871] |
| Unemployed, n (%) | 7 (41.2%) | 10 (58.8%) | -0.316 | 0.729 | [0.189, 2.817] | 21 (72.4%) | 8 (27.6%) | 2.106 | 0.182 | [0.705, 6.294] | 12 (80.0%) | 3 (20.0%) | 4.377 | 0.055 | [0.971, 19.724] |

*Notes*. * *p* < 0.050, ** *p* < 0.010. Univariate linear regressions/logistic regressions were conducted with financial deprivation status (deprived vs not deprived) and five potential confounding variables (DUP, years of education, schizophrenia diagnosis, place of birth, type of housing) and the baseline score of the variable as independent variables. Clinical, functional, and neurocognitive outcomes at 4 years were included as dependent variables. All assessments were administered by trained research assistants who underwent intensive training in the use of study instruments. ^a^ For age group 3: >42 years old (n=71), the dependent variable SAPS Inappropriate affect is a constant and regression statistics cannot be computed.

*Abbreviations*. PANSS - Positive and Negative Syndrome Scale, SAPS - Scale for Assessment of Positive Symptoms, SANS - Scale for Assessment of Negative Symptoms, CDSS - Calgary Depression Scale for Schizophrenia, MCQ - Medication Compliance Questionnaire, UKU - Udvalg for Kliniske Undersøgelser, SOFAS - Social and Occupational Functioning Scale, RFS - Role Functioning Scale, SF-12 - 12-item Short-Form Survey, MWCST - Modified Wisconsin Card Sorting Test.

**Reference**

1. Census and Statistics Department. Hong Kong poverty situation report 2012. 2012. https://www.commissiononpoverty.gov.hk/pdf/2012_Poverty_Situation_Eng.pdf.
